# Genetic Modification of Closely Related *Candida* Species

**DOI:** 10.3389/fmicb.2019.00357

**Published:** 2019-03-19

**Authors:** Eugenio Mancera, Corey Frazer, Allison M. Porman, Susana Ruiz-Castro, Alexander D. Johnson, Richard J. Bennett

**Affiliations:** ^1^Departamento de Ingeniería Genética, Centro de Investigación y de Estudios Avanzados del Instituto Politécnico Nacional, Unidad Irapuato, Irapuato, Mexico; ^2^Department of Molecular Microbiology and Immunology, Brown University, Providence, RI, United States; ^3^Department of Microbiology and Immunology, University of California, San Francisco, San Francisco, CA, United States

**Keywords:** *Candida dubliniensis*, *Candida tropicalis*, genetic modification, mNeonGreen, mScarlet

## Abstract

Species from the genus *Candida* are among the most important human fungal pathogens. Several of them are frequent commensals of the human microbiota but are also able to cause a variety of opportunistic infections, especially when the human host becomes immunocompromised. By far, most of the research to understand the molecular underpinnings of the pathogenesis of these species has focused on *Candida albicans*, the most virulent member of the genus. However, epidemiological data indicates that related *Candida* species are also clinically important. Here, we describe the generation of a set of strains and plasmids to genetically modify *C. dubliniensis* and *C. tropicalis*, the two pathogenic species most closely related to *C. albicans. C. dubliniensis* is an ideal model to understand *C. albicans* pathogenesis since it is the closest species to *C. albicans* but considerably less virulent. On the other hand, *C. tropicalis* is ranked among the four most common causes of infections by *Candida* species. Given that *C. dubliniensis* and *C. tropicalis* are obligate diploids with no known conventional sexual cycle, we generated strains that are auxotrophic for at least two amino acids which allows the tandem deletion of both alleles of a gene by complementing the two auxotrophies. The strains were generated in two different genetic backgrounds for each species — one for which the genomic sequence is available and a second clinically important one. In addition, we have adapted plasmids developed to delete genes and epitope/fluorophore tag proteins in *C. albicans* so that they can be employed in *C. tropicalis*. The tools generated here allow for efficient genetic modification of *C. dubliniensis* and *C. tropicalis*, and thus facilitate the study of the molecular basis of pathogenesis in these medically relevant fungi.

## Introduction

**Figure 1:**
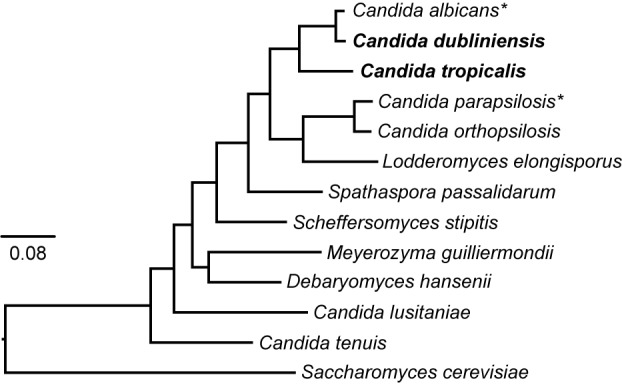
Phylogeny of the CTG clade. The phylogenetic relationships of the species belonging to the clade that has been traditionally known as the “CTG clade” and whose genome has been sequenced is shown. There are related ascomycete clades that also translate the CTG codon in alternative ways and it has therefore been proposed to name the clade CUG-Ser1 (Krassowski et al., 2018). The tree was generated by the Candida Gene Order Browser (CGOB) from 100,000 informative amino acid positions shared by all species as described (Maguire et al., 2013). *Candida dubliniensis* and *C. tropicalis* are shown in bold and asterisks denote species for which there are large collections of gene-deletion mutants. The tree was rooted using the budding yeast (*Saccharomyces cerevisiae*) which is a sister species of the clade. *C. glabrata* is not shown in the tree, but it is more closely related to *S. cerevisiae* than to CTG species.

Fungi from the genus *Candida* are a heterogeneous group of ascomycete yeasts. Although the human infections caused by *Candida* species (candidiasis) have been a subject of study since ancient Greece, it was not until 1923 that the name *Candida albicans* was proposed for the first member of the genus (Lynch, 1994). Nowadays, the genus comprises more than 160 species that were grouped in part because no clearly defined sexual cycle was identified (Turner and Butler, 2014). Therefore, the genus is polyphyletic and quite diverse. In addition, due to recent improvements and standardization in fungal taxonomy, many *Candida* species are being renamed (Gabaldon et al., 2016). More than 30 species of *Candida* have been identified as the causative agent of candidiasis. However, around 95% of the infections are caused by only four species: *C. albicans*, *C. glabrata*, *C. parapsilosis*, and *C. tropicalis* (Turner and Butler, 2014; Gabaldon et al., 2016). These species, except for *C. glabrata*, belong to a clade whose members translate the CTG codon as serine instead of leucine (Figure 1) (Butler et al., 2009). Although this clade has been traditionally known as the “CTG clade,” recent findings have shown that there are related clades of ascomycetes that also translate the CTG codon in non-standard ways (Krassowski et al., 2018). In addition to the three CTG clade species mentioned above, the CTG clade comprises other species that are rarer etiological disease agents such as *Candida dubliniensis*, *C. guilliermondii*, *C. lusitaniae*, and *C. auris*, with the latter associated with multidrug resistant infections (Moran et al., 2011; Gabaldon et al., 2016; Sears and Schwartz, 2017). Overall, the CTG clade encompasses most of the pathogenic species of *Candida*.

Pathogenic species of *Candida* are frequent commensals of the human microbiota. Some estimates suggest that as much as 70% of the adult human population is a carrier of some kind of yeast in the gastrointestinal tract, most often *Candida* species (Schulze and Sonnenborn, 2009). These species are able to asymptomatically colonize many human tissues (Lynch, 1994) with the gastrointestinal and genitourinary tracts of healthy individuals being especially common niches. However, under specific circumstances such as imbalances of the immune system or the local microbiota, *Candida* species are able to cause a variety of diseases, from superficial mucosal infections to life-threatening systemic conditions. In cases of hematogenously disseminated candidiasis, the mortality rates are as high as 40% (Nobile and Johnson, 2015). The incidence of diseases caused by *Candida* species has increased since the 1980s, especially of nosocomial infections. Although such an increase could partially be explained by better detection methods, it is also attributed to advances in medical practices. Therapies that alter the immune system, that allow survival of immunocompromised patients, or that involve the implantation of medical devices have broadened the impact of candidiasis in the human population (Gabaldon et al., 2016).

The frequency of infections caused by each *Candida* species varies depending on the geographical region, although *C. albicans* is consistently the most frequent cause of candidiasis being associated with more than 70% of cases in some regions (Turner and Butler, 2014). For this reason, most of the research to understand the molecular mechanisms responsible for the pathogenesis of these species has focused on *C. albicans*. Over the years, several strategies have been developed to genetically modify *C. albicans.* The most common strategy relies on homologous recombination and involves parental strains with two or more amino acid auxotrophies. The two alleles of a gene can thus be deleted in tandem by performing transformations with the two corresponding nutritional markers (Noble and Johnson, 2005). This strategy overcomes the intrinsic difficulties of genetically modifying this species given that it is diploid with no known conventional sexual cycle. Using such a strategy, several collections of gene deletion mutants have been generated in *C. albicans* (Homann et al., 2009; Noble et al., 2010). Approaches that employ counterselectable or recyclable markers such as the *SAT1* flipper system have also been instrumental for disrupting genes in *C. albicans*, especially in clinical isolates for which no auxotrophic strains have been generated (Wilson et al., 2000; Reuß et al., 2004). These methods are more time consuming since they involve an extra experimental step to remove the marker before the second allele can be targeted for deletion.

More recently, a number of approaches to genetically modify *C. albicans* using CRISPR-mediated systems have been developed (Vyas et al., 2015; Min et al., 2016; Grahl et al., 2017; Huang and Mitchell, 2017; Ng and Dean, 2017; Nguyen et al., 2017). While promising, CRISPR-mediated systems can also benefit from using auxotrophic strains that have previously been generated. For example, a recent CRISPR-Cas9 system developed for *C. albicans* involves insertion of a cassette at the *LEU2* locus, which results in an auxotrophic strain when utilizing a heterozygous *LEU2/*Δ*leu2* strain. After the target locus has been modified by CRISPR, restoration of the ability to grow in medium lacking leucine is used to select for cells that have lost the CRISPR-Cas9 cassette due to recombination at the *LEU2* locus (Nguyen et al., 2017). Transposon-mediated genetic modifications have also been used to disrupt genes in *C. albicans* to study gene essentiality in bulk (Uhl et al., 2003; Segal et al., 2018). Overall, the use of auxotrophic strains with their corresponding nutritional markers has been the strategy of choice to generate large collections of mutants, and is still the method most routinely used to generate gene-knockout strains in *C. albicans*.

Recent international surveys have asserted the medical importance of other species aside from *C. albicans*, especially in some geographic regions. For example, the incidence of *C. glabrata* and *C. parapsilosis* is particularly high in Europe and the Americas, respectively. In addition, these surveys have suggested that the incidence of these other species relative to *C. albicans* is increasing over time (Turner and Butler, 2014). Although this observation can be influenced by improvements in the methods to distinguish different *Candida* species, it could also be associated with the widespread use of antifungal drugs and the increased drug resistance of these species compared to *C. albicans* (Turner and Butler, 2014; Gabaldon et al., 2016). Given the interest in *C. glabrata* and *C. parapsilosis*, collections of gene deletion mutants have been generated using similar genetic approaches to those used for *C. albicans* (Holland et al., 2014; Schwarzmuller et al., 2014).

*Candida tropicalis* is one of the four most important causes of candidiasis worldwide. Its incidence is especially high in Africa and the Middle East and several strains are naturally resistant to fluconazole, the most commonly used antifungal drug (Turner and Butler, 2014; Gabaldon et al., 2016). A number of groups have now reported the generation of gene deletion mutants in this diploid species. Common strategies include the use of dual auxotrophic strains with corresponding genetic markers as in *C. albicans*, or a combination of single amino acid auxotrophic strains and drug resistance markers to delete the two alleles of a gene (Mancera et al., 2015; Anderson et al., 2016; Zhang et al., 2016; Zheng et al., 2017). In addition, strategies using counterselectable or recyclable markers have also been used to disrupt genes in *C. tropicalis*, especially when auxotrophic strains are not available for the isolate of interest (Mancera et al., 2015; Anderson et al., 2016; Wang et al., 2018). However, no large collections of gene-deletion strains are available. A variety of genetic backgrounds have been used in *C. tropicalis* and the genome sequence for only one of them is available to date.

In addition to the three CTG species mentioned above, *C. dubliniensis* has drawn considerable attention in recent years. Even though the incidence of this species as a cause of candidiasis is low, phylogenetically it is the closest known species to *C. albicans* (Sullivan et al., 2005) to the extent that they are able to mate with one another (Pujol et al., 2004). It has been estimated that these species diverged from a common ancestor around 20 million years ago and past epidemiological surveys may have often mistaken *C. dubliniensis* as *C. albicans* (Moran et al., 2012). Due to its evolutionary proximity but attenuated virulence, *C. dubliniensis* is being used as a model to identify the species-specific genetic determinants that underlie *C. albicans* pathogenicity. *C. dubliniensis* genes have been targeted using reusable drug-resistant markers developed for *C. albicans* as well as the *URA3* counterselectable marker (Staib et al., 2001). However, the use of *URA3* as a genetic marker is known to affect virulence and chromosomal stability in *C. albicans* and so is now avoided where possible (Staab and Sundstrom, 2003; Noble and Johnson, 2005).

In this work, we describe a set of strains and plasmids that allow for the generation of gene deletion mutants in *C. dubliniensis* and *C. tropicalis*, the two species most closely related to *C. albicans* (Figure 1). The strains are auxotrophic for different combinations of histidine, leucine and arginine. These three amino acid biosynthetic genes have been shown to be dispensable for *C. albicans* in the tail vein mouse model of disseminated infection and in the gastrointestinal mouse model of commensalism (Noble and Johnson, 2005; Pande et al., 2013). Gene deletion cassettes carrying the missing genes can be easily generated by fusion PCR. This is done from a set of plasmids that contain the *C. albicans HIS1*, *LEU2*, and *ARG4* cloned genes and which complement the auxotrophies of the strains. Since these markers are not the *C. dubliniensis* or *C. tropicalis* genes, the probability of insertion into the endogenous loci is diminished, thereby increasing the efficiency of recombination at the intended gene target. The auxotrophic strains have been generated in two different genetic backgrounds for each species. For both species, one of the backgrounds is the one whose genome has been sequenced to date. The other background has been used for molecular studies in these species including those by our groups. In addition, we describe modification of the *SAT1* flipper system developed for *C. albicans* so that it can be more efficiently used in *C. tropicalis.* Similarly, we have adapted *C. albicans* vectors to fuse genes with an epitope tag or a fluorescent protein for use in *C. tropicalis.* Overall, the strategies described here allow for the rapid construction of targeted gene deletions in *C. dubliniensis* and *C. tropicalis*, as well as other common genetic manipulations. This will facilitate the generation of collections of strains to better understand the molecular basis of pathogenesis in these medically important fungi.

## Materials and Methods

### Generation of Auxotrophic Strains

Auxotrophic strains were generated using the *SAT1*-flipping strategy (Sasse and Morschhauser, 2012). For the deletion of amino acid biosynthetic genes in *C. dubliniensis*, the *SAT1* reusable cassette was PCR amplified from the plasmid pSFS2A with primers containing ∼65 bp of sequence identical to the 5′ and 3′ regions immediately up and downstream of the *HIS1*, *LEU2*, and *ARG4* open reading frames (ORFs; primer pairs EMO001/EMO002, EMO003/EMO004 and EMO005/EMO006, respectively; Supplementary Table S1). pSFS2A is a plasmid derived from pSFS2 (Reuß et al., 2004) that contains the *SAT1* reusable cassette in the backbone of vector pBC SK+ instead of pBluescript II KS and that was kindly provided by Joachim Morschhauser (U. Würzburg). The cassettes were transformed into the CD36 and Wü284 wild type *C. dubliniensis* strains by electroporation as previously described (Kohler et al., 1997; Staib et al., 2001). For selection of transformants, cells were grown on YEPD medium plates containing 400 μg/ml of nourseothricin (NAT) for 48 h at 30°C. Correct integration of the *SAT1* cassette was confirmed by colony PCR of the 5′ and 3′ junctions. For the excision of the *SAT1* cassette cells were grown overnight at 30°C in YEP medium containing 2% maltose and then plated on YEPD plates containing 50 μg/ml NAT. After 24 h of growth at 30°C, small colonies were selected and further verified for the loss of NAT resistance by growth on YEPD + 400 μg/ml NAT plates for 48 h at 30°C, as has been described previously (Sasse and Morschhauser, 2012; Porman et al., 2013). Six successive rounds of gene deletion/flipping were needed to obtain the triple auxotrophic mutant. In the final strain, the loss of the three genes was confirmed by PCR with primers that anneal inside the ORF of each gene and by the inability of the strains to grow in synthetic defined (SD) media lacking histidine, leucine or arginine.

For the generation of the equivalent auxotrophic strains in *C. tropicalis*, we first attempted to use the *SAT1*-flipping strategy amplifying the deletion cassette with long oligonucleotides that have the 5′ and 3′ homology regions in the same way as for *C. dubliniensis*. Although we did obtain transformants using this strategy, the integration of the deletion cassette was not in the correct locus. To increase the specificity of the recombination process in this species we used longer homology arms flanking the *SAT1* cassette. For this purpose, ∼900 bp regions flanking the *HIS1*, *LEU2*, and *ARG4* ORFs were PCR amplified from genomic DNA and cloned adjacent to the *SAT1* cassette in the pSFS2A or pEM008 plasmids (see below for the description of pEM008 and Supplementary Table S1 for primers used). Preparation of the deletion cassettes for transformation was done either by digesting with the outermost restriction enzymes (see Supplementary Table S1), or by PCR using oligos that anneal to the cloned homology arms as a template. Transformation was performed in the MYA-3404 and AM2005/0093 *C. tropicalis* strain backgrounds, and selection of transformants and verification of correct integration was done as for *C. dubliniensis*. Flipping out of the *SAT1* marker when using the pSFS2A cassette was achieved by growing *C. tropicalis* cells for 2 days at 30°C in either liquid or solid YEP medium containing 2% maltose (Porman et al., 2013). Alternatively, flipping out of the markers coming from the pEM008 plasmid involved growing cells overnight at 30°C in either YNB medium with 2% casamino acids (Chauvel et al., 2012) or in synthetic medium containing 2% succinate (Gerami-Nejad et al., 2004). Cells were subsequently screened in YEPD supplemented with 400 μg/ml NAT plates for the loss of NAT resistance before proceeding to delete the second allele. As for *C. dubliniensis*, the absence of the gene was also verified by colony PCR with primers that amplify part of the ORF and by the inability of the strains to grow in SD media lacking the corresponding amino acid. The details of all generated strains are provided in Table 1.

**Table 1:** *C. dubliniensis* and *C. tropicalis* strains used in this study.

Name	Species	Background	Genotype	MTL	Source
CEM002	*C. dubliniensis*	CD36	*WT*	a/alpha	Derek Sullivan
CEM035	*C. dubliniensis*	CD36	*his1*Δ*::FRT/his1*Δ*::FRT*	a/alpha	This Study
CEM055	*C. dubliniensis*	CD36	*his1*Δ*::FRT/his1*Δ*::FRT Ieu2Δ::FRT/leu2*Δ*::FRT*	a/alpha	This Study
CEM074	*C. dubliniensis*	CD36	*his1*Δ*::FRT/his1*Δ*::FRT leu2Δ::FRT/leu2*Δ*::FRT arg4Δ::FRT/arg4Δ::FRT*	a/alpha	This Study
CEM158	*C. dubliniensis*	CD36	*leu2*Δ*::FRT/leu2Δ::FRT*	a/alpha	This Study
CEM160	*C. dubliniensis*	CD36	*arg4*Δ*::FRT/arg4Δ::FRT*	a/alpha	This Study
CEM003	*C. dubliniensis*	Wü284	*WT*	a/alpha	Joachim Morschhäuser
CEM041	*C. dubliniensis*	Wü284	*his1*Δ*::FRT/his1*Δ*::FRT*	a/alpha	This Study
CEM072	*C. dubliniensis*	Wü284	*his1*Δ*::FRT/his1*Δ*::FRT Ieu2Δ::FRT/leu2*Δ*::FRT*	a/alpha	This Study
CEM091	*C. dubliniensis*	CD36	*his1*Δ*::HIS1 Calb/his1*Δ*::FRT leu2*Δ*::LEU2 Calb/leu2Δ::FRT arg4Δ::FRT/arg4Δ::FRT*	a/alpha	This Study
CEM146	*C. dubliniensis*	CD36	*his1*Δ*::HIS1 Calb/his1*Δ*::FRT leu2*Δ*::LEU2 Calb/leu2*Δ*::FRT arg4Δ::ARG4 Calb/arg4Δ::FRT*	a/alpha	This Study
CEM148	*C. dubliniensis*	Wü284	*his1*Δ*::FRT/his1*Δ*::FRT leu2*Δ*::FRT/leu2*Δ*::FRT arg4Δ::FRT/arg4Δ::FRT*	a/alpha	This Study
CEM162	*C. dubliniensis*	Wü284	*leu2*Δ*::FRT/leu2Δ::FRT*	a/alpha	This Study
CEM164	*C. dubliniensis*	Wü284	*arg4*Δ*::FRT/arg4Δ::FRT*	a/alpha	This Study
CEM010	*C. tropicalis*	MYA3404	*WT*	a/alpha	ATCC
CEM206	*C. tropicalis*	MYA3404	*leu2*Δ*::FRT/leu2*Δ*::FRT*	a/alpha	Mancera et al., 2015
CSR001	*C. tropicalis*	MYA3404	*leu2*Δ*::FRT/leu2*Δ*::FRT his1*Δ*::FRT/his1Δ::FRT*	a/alpha	This Study
CEM300	*C. tropicalis*	MYA3404	*leu2*Δ*::LEU2 Calb/leu2*Δ*::FRT*	a/alpha	This Study
CAY4599	*C. tropicalis*	AM2005/0093	*WT*	a/a	Jacobsen et al., 2008
CAY3460	*C. tropicalis*	AM2005/0093	*leu2*Δ*::FRT/leu2*Δ*::FRT*	a/a	This Study
CAY3484	*C. tropicalis*	AM2005/0093	*his1*Δ*::FRT/his1*Δ*::FRT*	a/a	This Study
CAY3762	*C. tropicalis*	AM2005/0093	*his1*Δ*::FRT/his1*Δ*::FRT arg4*Δ*::FRT/arg4*Δ*::FRT*	a/a	This Study
CAY3763	*C. tropicalis*	AM2005/0093	*his1Δ::FRT/his1*Δ*::SAT1 leu2*Δ*::FRT/leu2*Δ*::FRT*	a/a	This Study
CAY3766	*C. tropicalis*	AM2005/0093	*leu2Δ::FRT/leu2*Δ*::FRT arg4*Δ*::FRT/arg4*Δ*::FRT*	a/a	This Study
CAY10231	*C. tropicalis*	AM2005/0093	*EFG1/EFG1-mScarlet*	a/a	This Study
CAY10233	*C. tropicalis*	AM2005/0093	*EFG1/EFG1-mNeonGreen*	a/a	This Study
CAY10139	*C. tropicalis*	AM2005/0093	*WOR1/WOR1-mNeonGreen*	a/a	This Study

### Generation of Plasmids Carrying Nutritional and Drug-Resistance Markers

To clone the *C. albicans HIS1*, *LEU2*, and *ARG4* genes for use as nutritional markers these genes were PCR amplified with their corresponding promoter and terminator using genomic DNA from the wild type SC5314 strain (see Supplementary Table S1 for primers). Each gene was cloned in the pCR-BluntII-TOPO vector using the Zero Blunt TOPO PCR cloning kit (Invitrogen) following the protocol described by the manufacturer (see Table 2 for plasmid names). The *SAT1* and *CaHygB* genes were PCR amplified from plasmids pSFS2A (Reuß et al., 2004) and pYM70 (Basso et al., 2010), respectively (see Supplementary Table S1 for primers). These genes were cloned in the pCR2.1-TOPO vector using the TOPO TA Cloning Kit (Invitrogen) using the manufacturer’s protocol. All cloned genes were verified by sequencing and the name and details of each resulting plasmid (including the GeneBank accession numbers) are shown in Table 2.

**Table 2:** Plasmids to genetically modify *C. dubliniensis* and *C. tropicalis*.

Name	Description	Antibiotic resistance	GB accession no.
pEM001	pCR-BluntII-TOPO with *C. albicans HIS1* marker	Kanamycin	MK425745
pEM002	pCR-BluntII-TOPO with *C. albicans LEU2* marker	Kanamycin	MK425746
pEM003	pCR-BluntII-TOPO with *C. albicans ARG4* marker	Kanamycin	MK425747
pEM004	pSFS2A with *C. tropicalis MAL2* promoter	Chloramphenicol	MK425748
pEM008	pSFS2A with *C. tropicalis PCK1* promoter instead of the *MAL2* (Figure 3)	Chloramphenicol	MK431394
pEM010	pSFS2A with *C. albicans PCK1* promoter instead of the *MAL2* (Figure 3)	Chloramphenicol	MK431395
pEM018	pEM008 with 13x MYC tag (Figure 3)	Chloramphenicol	MK431396
pEM019	pEM010 with 13x MYC tag (Figure 3)	Chloramphenicol	MK431397
pEM021	pCR2.1-TOPO with *HygB* marker from pYM70	Kanamycin/Ampicillin	MK431398
pEM025	pCR2.1-TOPO with *SAT1* marker from pSFS2A	Kanamycin/Ampicillin	MK431399
pSFS2A-mNeonGreen	pSFS2A with *C. albicans*-optimized mNeonGreen tag (Figure 3)	Chloramphenicol	MK431400
pSFS2A-mScarlet	pSFS2A with *C. albicans*-optimized mScarlet tag (Figure 3)	Chloramphenicol	MK427053

### Gene Deletion Strategy and Reintegration of Nutritional Markers

**Figure 2:**
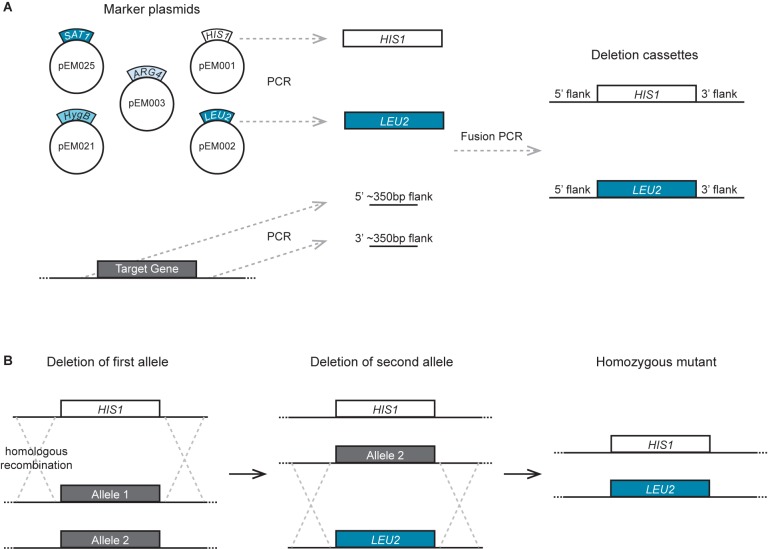
Strategy to generate gene deletion mutants in *C. dubliniensis* and *C. tropicalis.* **(A)** Depiction of the procedure to generate the deletion cassette. The markers are amplified by PCR using as a template one of the plasmids carrying the nutritional markers or the drug-resistant markers. ∼350 bp flanking regions of the target gene are also amplified by PCR using genomic DNA as a template. The three fragments are combined by fusion PCR to generate the deletion cassettes. **(B)** Scheme of the procedure to delete the two alleles of the gene of interest by transformation and homologous recombination using the deletion cassettes. Each allele is deleted in tandem using a different nutritional marker so that the markers do not need to be eliminated before targeting the second allele.

The overall methodology used to delete genes using the auxotrophic strains and markers described here is the same as that previously described for *C. albicans* (Figure 2) (Noble and Johnson, 2005; Hernday et al., 2010). Gene deletion cassettes were generated by fusion PCR of three fragments: the sequence coding for the nutritional marker, and ∼350 bp sequences flanking the 5′ and 3′ ends of the ORF to be deleted. The PCR amplification of the *HIS1*, *LEU2*, and *ARG4* nutritional markers was performed using plasmids pEM001, pEM002, and pEM003 as a template, respectively, with primers EMO126 and EMO622. These primers have a region that anneals to the TOPO vectors where the markers are cloned, and a second region that anneals to the primers used for the amplification of the flanks. A specific 20 bp sequence that functions as a “barcode” can be introduced to facilitate the identification of each gene deletion mutant. These barcode sequences can be included as part of the synthesis of primer EMO622 between the sequences GCAGGGATGCGGCCGCTGAC and AGCTCGGATCCACTAGTAACG as has been described (Noble and Johnson, 2005). PCR amplification of the flanks of the gene to be deleted was performed using *C. dubliniensis* or *C. tropicalis* genomic DNA as a template, together with primers designed to amplify the ∼350 bp region upstream and downstream of the ORF. The following two sequences were included at the 5′ end of the reverse primer for the 5′ flank amplification and the forward primer of the 3′ flank amplification, respectively: CACGGCGCGCCTAGCAGCGG and GTCAGCGGCCGCATCCCTGC. These sequences allow annealing of the flanks and the auxotrophic markers amplified with primers EMO126 and EMO622 for the fusion PCR reaction. Fusion PCR was performed as described (Hernday et al., 2010) by mixing the three PCR fragments in one final PCR reaction together with the forward primer used to amplify from the 5′ flank and the reverse primer used to amplify from the 3′ flank.

Generation of the deletion cassettes with the *SAT1* and *CaHygB* drug-resistance markers was performed as for the auxotrophic markers, but using the plasmids pEM025 and pEM021, respectively, as PCR templates. Similarly, the markers for *C. tropicalis* transformations with the markers from *C. maltosa* and *C. dubliniensis* that were originally used for *C. albicans* were PCR amplified from previously described plasmids (Noble and Johnson, 2005). These plasmids are pSN40 (*C. maltosa LEU2*), pSN52 (*C. dubliniensis HIS1*), and pSN69 (*C. dubliniensis ARG4*).

The products of fusion PCR reactions were purified and concentrated using a QIAGEN MinElute PCR Purification Kit, and between 0.5 and 2.5 μg of DNA were transformed by electroporation as described (Kohler et al., 1997; Staib et al., 2001). Selection of transformants was performed by growing cells on SD medium lacking the corresponding amino acid, or on YEPD plates supplemented with 400 μg/ml NAT or 500 μg/ml hygromycin B. Verification of correct integration into the genome was performed by colony PCR of the 5′ and 3′ junctions. In addition, after deleting the second allele, the absence of the target gene was verified by colony PCR with primers that amplify a region within the target ORF. Re-integration of the *C. albicans HIS1*, *LEU2*, and *ARG4* nutritional markers in *C. dubliniensis* and *C. tropicalis* was performed at the corresponding loci. The fusion PCR protocol described above was used to conjugate the *C. albicans* nutritional markers with ∼350 bp flanks of the *C. dubliniensis* and *C. tropicalis* nutritional genes. The corresponding primers are provided in Supplementary Table S1. Selection of transformants in SD media lacking the corresponding amino acid also provided proof of complementation of the auxotrophies by these markers in *C. dubliniensis* and *C. tropicalis*.

### Construction of an Improved *SAT1* Flipper Cassette for *C. tropicalis*

**Figure 3:**
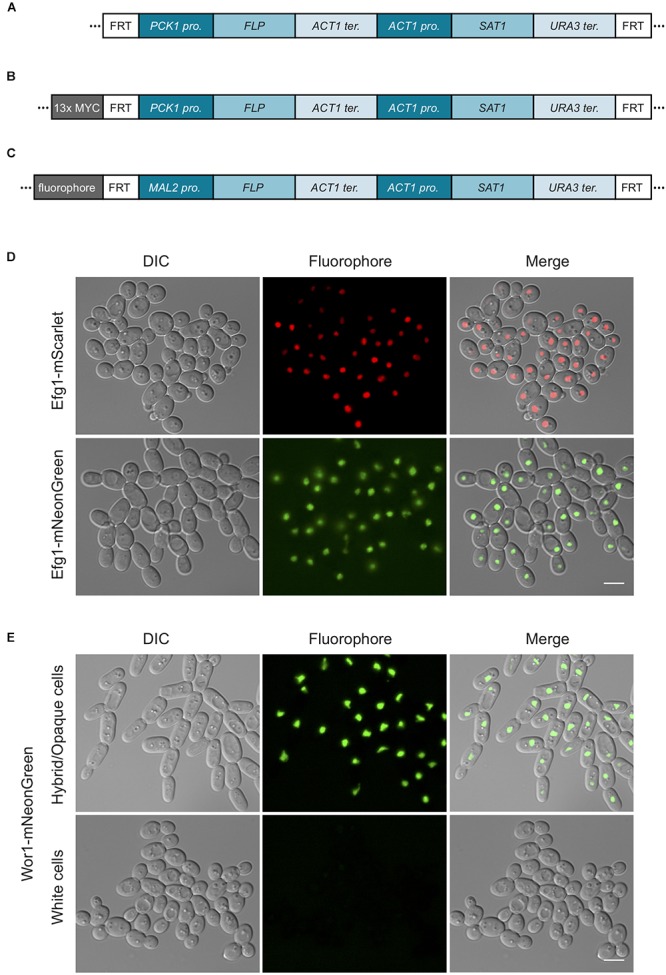
*SAT1*-flipper tools for *C. tropicalis*. **(A)** Depiction of the structure of the *SAT1* flipper cassette that can be used to delete genes in *C. tropicalis*. Here, the *FLP* gene is expressed under the control of the *PCK1* promoter. This cassette is cloned into plasmids pEM008 and pEM010 (Table 2). **(B)** Illustration of the structure of the cassette to C-terminally tag genes with a 13x MYC epitope tag that allows for rapid recycling of the *SAT1* marker. This cassette is cloned into plasmids pEM018 and pEM019 (Table 2). For cassettes in **(A,B)**, versions with either the *C. tropicalis* or the *C. albicans PCK1* promoter driving *FLP* were generated. **(C)** Depiction of the cassettes to C-terminally tag genes with either the mNeonGreen (pSFS2-mNeonGreen) or mScarlet (pSFS2-mScarlet) fluorophores. In all cassettes, recombination by the FLP recombinase results in the excision of the DNA region located between the two FRT sites from the genome. After recombination, only a single FRT site remains. The cassettes were generated in the pSFS2A plasmid that was itself constructed in a pBC SK+ plasmid backbone. The plasmid backbone, which is the same for all plasmids, is not shown. The sequences of the plasmids have been submitted to GeneBank and the accession numbers are provided in Table 2. pro., promoter; ter., terminator. **(D)** Micrographs showing the cellular localization of Efg1 C-terminal tagged with the fluorophores mScarlet or mNeonGreen. **(E)** Images showing the localization of the Wor1-mNeonGreen fusion in white and hybrid/opaque cells. The histogram of the mNeonGreen channel in white and hybrid/opaque cells is equivalent. In **(D,E)**, the left panels show cells by differential interference contrast microscopy (DIC), the central panels show the fluorophore signal, and the right panels the merged images. Scale bars, 5 μm.

In our experiments, the efficiency of recycling of the *SAT1* cassette derived from pSFS2A in *C. tropicalis* cells was much lower than that in *C. albicans.* To improve the efficiency of *SAT1* excision in *C. tropicalis*, we tested replacing the *C. albicans MAL2* promoter with its *C. tropicalis* homolog, or by using the *PCK1* promoter of *C. albicans* or *C. tropicalis* (Figure 3). The *C. albicans MAL2* promoter was previously cloned using BamHI and SalI sites in the plasmid pSFS2A (Reuß et al., 2004). However, there is a second SalI site in the *SAT1* ORF of plasmid pSFS2A that precludes re-using this site. To circumvent this issue, we used PCR fusion to combine a fragment containing the desired promoter and a 359 bp segment of the 5′ end of the *SAT1* gene from pSFS2A. The resulting PCR product was then cloned into pSFS2A using the BamHI site and an EcoRV site that is present within *SAT1*. The primer pairs used to PCR amplify the promoter and the *SAT1* fragment are the following: for the *C. tropicalis MAL2* promoter, EMO178 – EMO179 and EMO176 – EMO177; for the *C. tropicalis PCK1* promoter, EMO197 – EMO210 and EMO177 – EMO211; and for the *C. albicans PCK1* promoter, EMO242 – EMO243 and EMO177 – EMO244. For the fusion PCR, the following primer pairs were used: *C. tropicalis MAL2* promoter, EMO177 – EMO178; *C. tropicalis PCK1* promoter, EMO177 – EMO197; *C. albicans PCK1* promoter, EMO177 – EMO242. All primer sequences are shown in Supplementary Table S1. As shown in Table 2, the resulting vectors were named pEM004 (contains *C. tropicalis MAL2* promoter), pEM008 (contains *C. tropicalis PCK1* promoter), and pEM010 (contains *C. albicans PCK1* promoter). Flipping out of the *SAT1* cassette when using the *C. tropicalis MAL2* promoter was achieved by growing cells overnight at 30°C in YEP medium containing 2% maltose. When using the *C. albicans* or *C. tropicalis PCK1* promoters, cassette flipping was induced using either YNB medium with 2% casamino acids (Chauvel et al., 2012) or synthetic medium containing 2% succinate (Gerami-Nejad et al., 2004). Selection of cells that had lost the *SAT1* marker was done by growing isolated colonies in YEPD plates for 24 h and replica plating on YEPD plates supplemented with 400 μg/ml NAT.

To generate a vector that contains the cassette to epitope tag genes with an immunoprecipitable peptide and whose *SAT1* marker can be efficiently excised in *C. tropicalis*, we cloned the sequence coding for a 13x MYC tag into plasmids pEM008 and pEM010. As described above, the *SAT1* flippable cassette in these two plasmids uses the *PCK1* promoter to express the FLP gene (Figure 3). The 13x MYC tag sequence was amplified from the plasmid pADH34 (Hernday et al., 2010) using primers EMO249 and EMO250. This PCR product, that also contains the FRT site at the 3′ end of the MYC tag, was cloned into the BamH1 site of pEM008 and pEM010 that is immediately upstream of the *PCK1* promoter. Thus, induction of the FLP recombinase will excise the *SAT1* cassette leaving only the epitope tag at the C-terminus of the gene and the FRT site downstream of it. As shown in Table 2, the resulting plasmids were named pEM018 (*C. tropicalis PCK1* promoter) and pEM019 (*C. albicans PCK1* promoter).

Tagging of genes using these vectors was performed using a similar procedure to the one described for tagging *C. albicans* genes with the pADH34 vector (Hernday et al., 2010). For efficient transformations, homology regions for directing homologous recombination reactions were extended as was the case for most genetic modifications in *C. tropicalis*. We therefore generated ∼350 bp homology regions by fusion PCR of the DNA regions flanking the target gene together with the genetic marker cassette. The cassette was PCR amplified from vectors pEM018 and pEM019 using primers EMO623 and EMO624 (Supplementary Table S1). The primers used to extend the homology flanks were designed to amplify the ∼350 bp immediately up and downstream of the stop codon of the target gene, not including the stop codon. In addition, the reverse complement sequence of primer EMO623 (CCGTTAATTAACCCGGGGATCCG) was added to the 5′ end of the reverse primer to amplify the 5′ flank, and the reverse complement sequence of primer EMO624 (GATCCACTAGTTCTAGAGCGGCCGCC) was added to the 5′ end of the forward primer to amplify the 3′ flank. These two sequences allow the flanks to anneal to the tagging cassette for the fusion PCR. Fusion PCR was performed as before to combine the three PCR fragments, using the forward primer employed to amplify the 5′ flank and the reverse primer used to amplify the 3′ flank. The product of the fusion PCR was purified, concentrated and transformed by electroporation, as described above. Selection of transformants was performed on YEPD plates with 400 μg/ml NAT for 48 h at 30°C, and correct integration verified by colony PCR of junction regions. Flipping out of the *SAT1* cassette was performed as described above by growing cells in YNB medium with 2% casamino acids. Verification of gene tagging was performed by PCR amplification and Sanger sequencing of the ORF-tag junction.

### C-Terminal Tagging With Next-Generation Fluorophores

The ORFs of mNeonGreen or mScarlet-I fluorophores (Shaner et al., 2013; Bindels et al., 2017) were codon optimized for expression in *C. albicans* including a GGSG linker on the 5′ end, a stop codon, and flanking XhoI sites^1^. These sequences were cloned into pSFS2A which had been digested with XhoI and treated with calf intestinal phosphatase. Correct orientation of the insert was confirmed by restriction digest analysis and Sanger sequencing (Figure 3). Details of the resulting plasmids can be seen in Table 2 including GeneBank accession numbers. To tag *EFG1* and *WOR1* with the mNeonGreen or mScarlet-I fluorophores, the tagging cassette was amplified from plasmid pSFS2-mNeonGreen or pSFS2-mScarlet using primers with ∼80 bp homology sequences to the region immediately up and down of the corresponding ORF stop codon, excluding the stop codon itself (for primers see Supplementary Table S1). The cassette was then transformed by electroporation as described above and transformants were selected on YEPD + 400 μg/ml NAT plates for 48 h at 30°C. Correct integration of the fluorophore tag was verified by colony PCR of integration junctions. *WOR1* was tagged in cells in the white state, which were afterwards switched to opaque as previously described (Mancera et al., 2015). For visualization under the microscope, cells of all tagged strains were grown overnight in liquid Spider medium at room temperature. Cells were imaged using a Zeiss Observer.Z1 equipped with a CoolSnap HQ_2_ camera, Colibi 470 nm LED light source and Zeiss filter set 38 (GFP) and 45 (Texas Red).

## Results

### A Set of Strains and Plasmids to Genetically Modify *C. dubliniensis*

*Candida dubliniensis* is a member of the CTG clade that has only been isolated in the diploid form and for which no conventional sexual cycle has been identified. To be able to delete genes in this species, we generated parental strains that are auxotrophic for histidine, leucine and arginine (Table 1). These strains were generated by deleting the open reading frames (ORFs) of both alleles of the *HIS1*, *LEU2*, and *ARG4* genes employing the *SAT1*-flipping strategy previously used for gene deletions in *C. albicans* (Reuß et al., 2004). In these strains, two of the auxotrophies can be used to select for replacement of each allele of a given target gene with the corresponding amino acid biosynthetic gene. The third auxotrophy is convenient as it can be used to select for reintegration of the gene of interest to verify the phenotype of the mutant, as well as other genetic modifications. For the generation of deletion cassettes, the *HIS1*, *LEU2*, and *ARG4* genes of *C. albicans* were independently cloned with their endogenous promoters and terminators in the same plasmid backbone so that they can be amplified with a unique set of primers (Table 2). The amino acid sequence identity of the *HIS1*, *LEU2*, and *ARG4* genes between *C. albicans* and *C. dubliniensis* is 98, 97, and 99%, respectively. Despite the similarity of these genes at the amino acid level, the differences at the nucleotide level (90–94% identity) are expected to lower the probability that the amino acid biosynthetic markers will integrate back into the corresponding endogenous loci in *C. dubliniensis*.

As shown in Figure 2, the strategy presented here employs fusion PCR to join DNA fragments together to generate the deletion cassettes for transformation. PCR amplicons of ∼350 bp flanking the 5′ and 3′ ends of the target gene are fused to the sequence of the desired amino acid biosynthetic gene. The PCR-amplified deletion cassettes are then transformed into auxotrophic parental strains and selection performed in medium lacking the corresponding amino acid. The correct integration of the amino acid marker into the locus of interest is then verified by colony PCR. As a proof of concept, we integrated the *C. albicans HIS1*, *LEU2*, and *ARG4* genes into *C. dubliniensis* strains deleted for the corresponding endogenous genes. Importantly, all three *C. albicans* genes complemented for the auxotrophies in *C. dubliniensis*. *C. dubliniensis* strains carrying the three integrated *C. albicans* genes are also useful as a control to compare other gene deletion mutants using this method. Overall, the gene deletion strategy presented here is very similar to that successfully used to generate large collections of mutants in *C. albicans* and *C. parapsilosis*. We have successfully generated several gene deletion mutants in *C. dubliniensis* that will be described elsewhere (E. M. et al., manuscript in preparation). The two alleles of target genes were successfully deleted by two consecutive transformations, suggesting that the process to knockout these genes or the nutritional markers did not cause extensive aneuploidies, which is a possible side effect of genetically modifying these species.

The *C. dubliniensis* auxotrophic strains were generated in two different genetic backgrounds, CD36 and Wü284. CD36 is the type strain and is the only *C. dubliniensis* strain whose genome has been sequenced to date (Jackson et al., 2009). Wü284 on the other hand is the strain that has been most used to study *C. dubliniensis* at a molecular level. The first genetic modification systems in *C. dubliniensis* using the *URA3* marker were developed in this genetic background, and even the group that reported the sequence of CD36 used Wü284 for some phenotypic assays (Staib et al., 2001; Jackson et al., 2009). We generated the triple auxotrophic strains in both backgrounds as a starting point for gene deletion construction. As shown in Table 1, we also generated single auxotrophic strains for each amino acid and the double histidine/leucine auxotroph. All of these strains are available and will greatly facilitate genetic studies of *C. dubliniensis.*

### A Set of Strains and Plasmids to Genetically Modify *C. tropicalis*

As is the case for *C. dubliniensis*, no conventional sexual cycle or haploid forms are known for *C. tropicalis*. Thus, the same difficulties exist for genetic manipulation of the species as for other closely related members of the CTG clade. To facilitate genetic modification of *C. tropicalis*, we generated a set of strains that are auxotrophic for two of the three following amino acids: histidine, leucine and arginine (Table 1). As for *C. dubliniensis*, these parental strains were generated by deleting the ORFs of both alleles of *HIS1*, *LEU2*, and *ARG4* using a *SAT1*-flipping strategy. However, in contrast to strain construction in *C. dubliniensis*, deletion of the amino acid biosynthetic genes in *C. tropicalis* utilized longer homology arms for efficient targeting of the cassette into the genome. As described in detail in the Methods, ∼900 bp flanking sequences were cloned into the plasmid containing the *SAT1* marker to generate the deletion cassettes for the three genes. The observed differences in the integration efficiency between the two species may reflect dissimilarities in their DNA double-strand break repair mechanisms. For example, non-homologous end-joining (NHEJ) rather than homologous recombination could be the preferred repair pathway in *C. tropicalis* when the flanking homology regions are short.

The same strategy outlined for *C. dubliniensis* can be used to delete genes in auxotrophic *C. tropicalis* strains. The cloned *HIS1*, *LEU2*, and *ARG4* genes are used as templates for the generation of deletion cassettes which are transformed into the corresponding parental strains. We used either the amino acid biosynthetic genes from *C. albicans* as described above for *C. dubliniensis*, or *C. maltosa LEU2* and *C. dubliniensis HIS1* and *ARG4* that have been used for strain construction in *C. albicans*. The protein sequence identity between *C. tropicalis, C. albicans, C. maltosa*, and *C. dubliniensis* amino acid biosynthetic genes ranges between 86 and 99%, and all of the genes tested complemented the corresponding auxotrophy in *C. tropicalis*. The correct integration of the auxotrophic markers was verified by PCR of the DNA junctions between the deleted gene and the targeting cassette. In addition to the three nutritional markers described above, we also cloned the *SAT1* and *CaHygB* genes into a similar plasmid backbone (Table 2) and used these for providing resistance to nourseothricin and hygromycin B, respectively. These markers can be PCR amplified with the same set of primers used for the nutritional markers to generate gene deletion cassettes. We have successfully used both nutritional markers and the drug resistance markers in different combinations to effectively delete multiple genes in *C. tropicalis* (Mancera et al., 2015; Anderson et al., 2016; E. M. et al., manuscript in preparation). As with *C. dubliniensis*, the two alleles of different target genes were successfully deleted in tandem, which suggests that these strains do not contain aneuploid chromosomes.

The *C. tropicalis* auxotrophic strains were generated in MYA-3404 and AM2005/0093 genetic backgrounds. MYA-3404 is the strain whose genome has been sequenced (Butler et al., 2009), while AM2005/0093 is a strain that was isolated from urine in Colombia and has been phylogenetically placed close to MYA-3404 by multi-locus sequence typing (Jacobsen et al., 2008). However, AM2005/0093 was naturally homozygous for the mating type-like (*MTL*) locus and thus was one of several strains picked to study the molecular mechanisms that control phenotypic switching, as switching is *MTL*-regulated in *C. albicans* (Mancera et al., 2015; Anderson et al., 2016). We had previously reported some of the auxotrophic strains generated in the AM2005/0093 background. Overall, the strains generated in the two genetic backgrounds (Table 1) offer an ideal starting point for the generation of gene deletion mutants in *C. tropicalis*.

### A *SAT1*-Flipping Strategy to Efficiently Delete or Epitope Tag Genes in *C. tropicalis*

The *SAT1* flippable cassette was originally developed for facilitating genetic manipulation of the *C. albicans* genome (Reuß et al., 2004). It is a powerful genetic engineering tool for diploid species since it allows the deletion of both alleles of a target gene even in the absence of available nutritional markers. Several variations of the system have been constructed, but in general the system employs the FLP site-specific recombinase to act on two FLP recognition target sites (FRT) that are present in direct repeats flanking the cassette containing the selectable marker. The FLP recombinase catalyzes excision of the region between the FRT sites and removes the selection marker, thus allowing for repeated use of the same marker for other genetic modifications. In a recent version of the system, the FLP gene is conditionally expressed using the promoter of the *C. albicans MAL2* gene which is induced when cells are grown in media containing maltose as the carbon source (Sasse and Morschhauser, 2012). Overall, the system combines a drug-resistance marker that allows efficient selection of transformants with an effective system for recycling the marker.

The same *SAT1*-flipping strategy has also been used successfully in *C. dubliniensis* and *C. tropicalis* to delete genes and was used here for the generation of auxotrophic strains. Nevertheless, the FLP-mediated excision of the deletion cassette was much less efficient in *C. tropicalis* than in *C. albicans*. Even after several days of growth in maltose-containing media very few cells lost the *SAT1* marker, hindering the overall utility of this reusable system. The difficulties in *SAT1* recycling likely reflect poor induction of *FLP* using the *CaMAL2* promoter in *C. tropicalis* cells. We therefore first replaced the *CaMAL2* promoter with the endogenous version from *C. tropicalis*, but FLP-induced recombination was still rare. As a second strategy, we tested driving FLP expression from the promoter of the *C. tropicalis PCK1* gene or the *C. albicans PCK1* gene (Figure 3). The *PCK1* promoter has previously been used to regulate gene expression in *C. albicans* as it is induced by gluconeogenic carbon sources (Gerami-Nejad et al., 2004; Chauvel et al., 2012). We therefore tested *SAT1* recycling by growing cells in succinate or casamino acids as the carbon source. Using either the *C. albicans* or the *C. tropicalis PCK1* promoter we found that excision of *SAT1* in *C. tropicalis* was as efficient as that using the *MAL2* promoter-driven FLP flipper system in *C. albicans*. We have therefore engineered an efficient system for marker recycling in *C. tropicalis*.

The recyclable *SAT1* cassette has also been used as part of a construct to epitope tag genes in *C. albicans* (Hernday et al., 2010). For this purpose, *SAT1* is placed adjacent to the epitope tag so that nourseothricin can be used to select transformants, and the *FLP* gene is then induced to flip-out the *SAT1* cassette leaving only the epitope tag fused to the gene and an FRT site downstream of the tag. Removal of the *SAT1* cassette diminishes the potential for disruption of the untranslated region (UTR) to impact gene function. To adapt this system for *C. tropicalis*, we cloned a 13x MYC tag into the plasmids described above that use the *C. tropicalis* or *C. albicans PCK1* promoter to regulate *FLP* expression (Figure 3). When tested, this strategy allowed efficient excision of the *SAT1* marker after epitope tagging several genes. The results of the immunoprecipitation of these genes will be described elsewhere (E. M. et al., manuscript in preparation). It is important to note that genome-wide sequencing data from the immunoprecipitation experiments does not show any evidence of large-scale copy number variations in these strains, again consistent with them having euploid genomes.

### A Set of Plasmids to Tag *Candida* Genes With Next-Generation Fluorophores

It has not been trivial to use conventional fluorophores such as GFP to visualize the cellular localization of gene products in *C. tropicalis*. For example, although we are able to generate the appropriate genetically modified strains, a number of protein-GFP fusions or GFP reporters were not visible under the microscope, with the exception of highly expressed histone-GFP and histone-mCherry fusions (Porman et al., 2011). The yeast-enhanced GFP and YFP have also been expressed successfully from a strong constitutive promoter when not fused to a target gene (Defosse et al., 2018). To enable cell biological approaches in *C. tropicalis*, we engineered plasmids that contain the next-generation fluorophores mNeonGreen and mScarlet that had been codon-optimized for *C. albicans*^2^ (Figure 3). These plasmids can be used to generate gene-fluorophore fusions by amplifying the fluorophore cassette using oligonucleotides with sequence identity to the C-terminus of the gene of interest. The fluorophores were cloned in the *SAT1* flippable system so that transformants can be selected using nourseothricin, and the drug-resistance marker flipped out by induction of the *FLP* gene. As a proof of concept, we tagged the transcription factors Efg1 and Wor1 with mNeonGreen, and also tagged Efg1 with mScarlet. As observed in Figure 3, it was possible to visualize the cellular localization of Efg1 when fused to both fluorophores. Efg1 is a transcription factor that regulates morphological transitions in *C. tropicalis* including filamentation and white-opaque switching (Mancera et al., 2015). The Wor1-mNeonGreen fusion was also detected, and this protein was only visible in cells that were in the hybrid/opaque state and not visible in cells in the white state. This is in agreement with the role of this transcription factor in determining the cell’s phenotypic state (Porman et al., 2013; Anderson et al., 2016). The discrete localization of both transcription factors in the nucleus is consistent with their function as transcriptional regulators. We have therefore successfully engineered tools that can be used to tag proteins with next-generation fluorophores in order to improve the visualization of gene products in *C. tropicalis* cells.

## Discussion

Fungi from the genus *Candida* are among the most important fungal human pathogens. They are a common source of a variety of diseases and can cause opportunistic infections with high mortality rates. Most research has focused on understanding *C. albicans* pathogenicity since it is the most prevalent *Candida* pathogen. However, other members of the genus are of high medical relevance, especially in some regions of the world. In addition, modern medical practices, such as the prophylactic use of antifungal drugs, appear to be favoring the increase in prevalence of alternative *Candida* species.

Here, we report the generation of a broad set of tools for genetic manipulation of *C. dubliniensis* and *C. tropicalis*, the two most closely related species to *C. albicans*. The strategies described here overcome the intrinsic difficulties of genetically engineering in diploid species that do not undergo a conventional sexual cycle. The parental strains are auxotrophic for *HIS1*, *LEU2*, and/or *ARG4* nutritional markers that have been used in the past to construct large collections of gene deletion mutants in *C. albicans* and *C. parapsilosis*. We also generated vectors containing the *HIS1*, *LEU2*, and *ARG4* nutritional markers from *C. albicans*, as well as the *SAT1* and *CaHygB* drug resistance markers in a similar plasmid backbone. This allows the PCR amplification of all the markers with the same set of primers for generation of the deletion cassettes. Two different selectable markers can then be used to delete both alleles of the target gene without the need to recycle the marker between the two rounds of transformation. This improvement could be crucial when attempting to generate large collections of knockout strains. In addition, we have engineered plasmids to create epitope and fluorophore gene fusions in *C. tropicalis* that allow for the immunoprecipitation of proteins as well as the study of their cellular localization. These tools will be useful in the study of this species at both a cellular and molecular level.

In the process of generating strains, we observed two interesting differences between *C. dubliniensis* and *C. tropicalis* in terms of the ease with which they can be genetically modified. The first concerns the specificity of the homologous recombination process as short homology regions (∼60 bp) are enough to specifically target the locus of interest in *C. dubliniensis* as has been reported for *C. albicans* (Hernday et al., 2010). In contrast, genetic modification of *C. tropicalis* often needed longer homology regions (∼350 bp) for efficient gene deletion, although successful integration of tagging constructs has been achieved with homology regions of ∼80 bp (Porman et al., 2011). These differences could be due to how DNA double-strand breaks are repaired in these species, with non-homologous end joining (NHEJ) being used in *C. tropicalis* when the homology regions are short. The strategies presented here overcome this difficulty since the deletion cassettes are generated by fusion PCR of the marker allowing the use of long homology flanks.

A second major difference observed between *C. dubliniensis* and *C. tropicalis* concerned the efficiency of *SAT1* marker recycling when using the FLP recombinase under the control of the *MAL2* promoter. In *C. dubliniensis*, FLP-mediated site-specific recombination was efficient, as previously described in *C. albicans* (Reuß et al., 2004; Sasse and Morschhauser, 2012). In contrast, marker excision was infrequent in *C. tropicalis* following growth on maltose-containing medium when using the *CaMAL2* promoter. We tested replacing the *CaMAL2* promoter with the endogenous *C. tropicalis* promoter yet still observed low *SAT1* excision rates, which could indicate low expression of this promoter in *C. tropicalis*. However, we found that replacing the *MAL2* promoter with the *PCK1* promoter (from either *C. tropicalis* or *C. albicans*) led to efficient excision of the *SAT1* marker in *C. tropicalis* when cells were grown on a gluconeogenic carbon source. This established that low expression of the *FLP* gene from the *MAL2* promoter was the limitation, and may indicate differences in the pathways used by these two *Candida* species to metabolize maltose.

Overall, the genetic tools presented here for *C. dubliniensis* and *C. tropicalis* will facilitate genetic studies of *Candida* biology and pathogenicity. *C. dubliniensis* is an ideal model to understand *C. albicans* pathogenicity given its phylogenetic proximity yet attenuated virulence. *C. tropicalis* is a sister species of the previous two and is consistently ranked amongst the four most prevalent disease-causing *Candida* species. Together with *C. albicans* and *C. parapsilosis*, these species are also useful for evolutionary studies (Figure 1) as they span a considerable evolutionary range but are close enough phylogenetically to capture subtle changes between their genomes (Butler et al., 2009). The genetic modification tools presented here will further accelerate the establishment of these species as evolutionary models.

## Data Availability

The datasets generated for this study can be found in GenBank, MK425745, MK425746, MK425747, MK425748, MK431394, MK431395, MK431396, MK431397, MK431398, MK431399, MK431400, and MK427053.

## Author Contributions

AJ, AP, EM, and RB contributed to the conception and design of the study. AP, CF, EM, and SR-C constructed the strains and plasmids. EM wrote the first draft of the manuscript. EM and RB wrote sections of the manuscript. All authors contributed to manuscript revision, and read and approved the submitted version.

## Conflict of Interest Statement

The authors declare that the research was conducted in the absence of any commercial or financial relationships that could be construed as a potential conflict of interest.

## Footnotes

1 https://currentprotocols.onlinelibrary.wiley.com/doi/abs/10.1002/cpmc.76

2 https://currentprotocols.onlinelibrary.wiley.com/doi/abs/10.1002/cpmc.76
