# Corrigendum: Genetic Modification of Closely Related Candida Species

**DOI:** 10.3389/fmicb.2020.00713

**Published:** 2020-04-15

**Authors:** Eugenio Mancera, Corey Frazer, Allison M. Porman, Susana Ruiz-Castro, Alexander D. Johnson, Richard J. Bennett

**Affiliations:** ^1^Departamento de Ingeniería Genética, Centro de Investigación y de Estudios Avanzados del Instituto Politécnico Nacional, Unidad Irapuato, Irapuato, Mexico; ^2^Department of Molecular Microbiology and Immunology, Brown University, Providence, RI, United States; ^3^Department of Microbiology and Immunology, University of California, San Francisco, San Francisco, CA, United States

**Keywords:** *Candida dubliniensis*, *Candida tropicalis*, genetic modification, mNeonGreen, mScarlet

In the published article, there was an error in the affiliation of Eugenio Mancera. Instead of “1,2”, it should only be “1”.

The authors apologize for this error and state that this does not change the scientific conclusions of the article in any way. The original article has been updated.

